# 81 Juvenile RHUPUS syndrome: a case reports

**DOI:** 10.1093/rheumatology/keac496.077

**Published:** 2022-10-07

**Authors:** Ourida Gacem, Kahina Abba, Khaoula Tenkout, Salima Abbas, Saliha Guers, Mohamed Chouli, Moussa Achir, Mohamed Samir Ladj

**Affiliations:** Pediatrics Department Hospital Djillali Belkhenchir Birtraria Algiers, Algeria

**Keywords:** *Systemic lupus erythematosus*, *Juvenile idiopathic arthritis*, *RHUPUS syndrome*

## Abstract

**Background:**

RHUPUS syndrome is a rare association with rheumatoid arthritis and systemic lupus erythematosus (SLE) in adult patients. Its pediatric presentation is very rare and underdiagnosed. The mechanism is not well understood yet, but most theories accepted a real overlap between SLE and juvenile idiopathic arthritis. In children. Patients with Rhupus have clinical symptoms of SLE with positive ANA, anti-DNA or anti Sm associated with clinical symptoms of JIA.

**Objective:**

Illustrate an unusual presentation of lupus in children

**Methods:**

we present a case of juvenile rhupus syndrome; we describe the clinical presentation, the serological results, the diagnostic criteria for SLE (ACR 1997) and JIA (ILLA 2001) and the treatment installed.

**Case reports:**

We recently diagnosed Rhupus syndrome in an 11-year-old girl who presented with polyarthritis deformans of the bilateral joints of the wrists, hands and feet for 24 months. she also had an onset of profound asthenia, recurrent oral aphtosis and massive hair loss over the past few months. Initial investigation showed anaemia, increased erythrocyte sedimentation rate (ESR) and C-reactive protein (CRP). Radiographic examinations showed juxtra-articular osteopenia and chronic synovitis of the wrists. The autoimmune assessment was contributory with a positive rheumatoid factor (RF), antinuclear antibodies (ANA) (1/1000) and positive anti-Sm antibodies. Anticardiolipin and anti-RNP antibodies were negative. Our patient met >4 ACR criteria for SLE classification. She was treated with methotrexate and hydroxychloroquine, under close medical supervision in order to watch for the appearance of other organic damage to lupus disease, in particular renal and neurological. This type of joint damage is considered to be either lupus joint damage, lupus with chronic arthritis, or overlapping lupus with JIA. Children with Rhupus initially present with JIA and later develop lupus. Previous reports have shown female predominance, polyarticular involvement, non-erosive arthritis, and years of diagnostic wandering. Our patient had polyarthritis deformans with a two-year delay in diagnosis of SLE.

**Conclusion:**

Although rare, the infantile Rhupus syndrome must be evoked in front of a deforming arthropathy.

